# Etablierung eines deutschen ICCR-Datensatzes: Übersetzung und Einbindung von SNOMED CT am Beispiel der TUR-B

**DOI:** 10.1007/s00292-024-01398-3

**Published:** 2024-12-05

**Authors:** J. Dörenberg, C. J. Schmidt, T. Berlage, R. Knüchel-Clarke

**Affiliations:** 1 RWTH Aachen University, Templergraben 55, 52062 Aachen, Deutschland; 2 Institut für Pathologie, Uniklinik RWTH Aachen, Pauwelsstraße 30, 52074 Aachen, Deutschland; 3 Zentrum für Integrierte Onkologie Aachen Bonn Köln Düsseldorf (CIO ABCD), Standort Aachen, Aachen, Deutschland; 4 Klinik für Urologie und Kinderurologie, Uniklinik RWTH Aachen, Aachen, Deutschland; 5 Fraunhofer Institut für angewandte Informationstechnik, Sankt Augustin, Deutschland

**Keywords:** Data Literacy, Qualitätsmanagement, Interdisziplinarität, Uropathologie, Tumorpathologie, Data Literacy, Quality management, Interdisciplinarity, Uropathology, Tumour pathology

## Abstract

**Hintergrund:**

Für die Qualitätssicherung in der Pathologie ist die strukturierte Erfassung von Daten aus histopathologischen Befunden sowie die Interoperabilität dieser Daten von zentraler Bedeutung.

**Methodik:**

Zur Harmonisierung des Inhalts der Befunde hat die International Collaboration on Cancer Reporting (ICCR) standardisierte Datensätze erstellt. Diese liegen noch nicht flächendeckend auf Deutsch vor. Hier wird diese Lücke am Beispiel des Usecase der transurethralen Blasenresektion (TUR-B) adressiert.

**Ergebnisse:**

Wir beschreiben den Prozess der Etablierung der Datensätze durch Übersetzung, Einbindung von SNOMED-CT-Codes und Nutzung der Hierarchie in SNOMED zum Füllen der Felder des Datensatzes. Zusätzlich definieren wir Regeln zur Datenqualitätsprüfung anhand der TUR‑B.

**Diskussion:**

Mit diesem Beitrag haben wir beispielhaft eine deutsche Version der ICCR-Datensatz TUR‑B inklusive Mapping auf die SNOMED-CT-Terminologie geschaffen. Weitere Schritte sollten nun in der abstrakten Modellierung von Erkrankungen liegen, um das in SNOMED CT liegende Potenzial weiter auszuschöpfen.

**Zusatzmaterial online:**

Zusätzliche Informationen sind in der Online-Version dieses Artikels (10.1007/s00292-024-01398-3) enthalten.

## Abstract (en)

**Background:**

The structured recording of data from histopathological findings and their interoperability is critical for quality assurance in pathology.

**Materials and methods:**

To harmonize the content of the reports, the International Collaboration on Cancer Reporting (ICCR) has defined standardized datasets. These datasets are not yet available in German nationwide. This gap is addressed here using the transurethral bladder resection (TUR-B) dataset as a use case.

**Results:**

We describe the process of establishing the datasets by carrying out translation, mapping on SNOMED CT codes, and using SNOMED CTs hierarchy to fill dropdown menus. Furthermore, we identified rules for checking for self-consistency of reports by using the example of the TUR bladder.

**Discussion:**

With this article, we have created an example of a German version of the ICCR TUR‑B dataset including mapping to the SNOMED CT terminology. Further activities should include the definition of overarching cancer disease models to further exploit the potential of SNOMED CT.

## Hintergrund und Fragestellung

Histopathologische Befunde werden in Deutschland derzeit meist in Textform verfasst und enthalten – trotz Unterteilung in bspw. Makroskopie, Histologie und Gutachten – nur wenige strukturierte Elemente. Daher ist eine zuverlässige, computerbasierte (Daten‑)Qualitätsprüfung – als Ergänzung zum bereits etablierten Vier-Augen-Prinzip sowie der Diskussion in interdisziplinären Konferenzen – nur eingeschränkt möglich. Das Konzept des *Synoptischen Reportings *(SR; [1, 7]) adressiert diese Probleme.

Das Grundprinzip von SR ist dabei das Ersetzen von Fließtext durch Frage-Antwort-Paare – bspw. Frage: Invasionstiefe, Antwort: pT2 – sowie die Bindung der Frage und der Antworten an vorgegebene Terminologien oder definierte Wertebereiche. Der Vorteil von SR liegt in 2 Aspekten. Erstens erleichtert SR verschiedene Formen von Datenqualitätsprüfungen – bspw. Prüfung auf Widersprüche. Zweitens werden die Ärztinnen und Ärzte bei der Befunderstellung durch einen strukturierten Prozess geführt. Dies erleichtert die Ausbildung von Assistenzärztinnen und -ärzten und erhöht die Prozessqualität in der Pathologie.

Im Rahmen der Einführung von SR ist es erforderlich, den Inhalt histopathologischer Befunde zu standardisieren. Die *International Collaboration on Cancer Reporting (ICCR)* hat hier bereits eine Reihe standardisierter Datensätze für unterschiedliche Organe und Fragestellungen erarbeitet. Trotz vieler Aktivitäten in diesem Bereich – vgl. zum Beispiel [2–4] – existieren noch keine standardisierten deutschsprachigen Fassungen dieser Datensätze. Über die Übersetzung hinaus ist es im Rahmen von SR erforderlich, die Interoperabilität der Befunddaten sicherzustellen. Ein wesentlicher Schritt hierbei ist die Nutzung des Terminologiesystems *Standardised Nomenclature for Medicine (SNOMED CT)*. Die automatisiert durchführbare Kodierung der Befunddaten in SNOMED CT gewährleistet Alltagsgebräuchlichkeit sowie den internationalen Charakter des Datensatzes im Sinne einer Interoperabilität der Daten bspw. im Rahmen des European Health Data Space.

Der vorliegende Beitrag beschreibt anhand des Usecase der *transurethralen Blasenresektion (TUR-B)* und des entsprechenden ICCR-Datensatzes [5] ein methodisches Vorgehen, um die bisher nicht auf Deutsch verfügbaren ICCR-Datensätze zu übersetzen. Gleichzeitig werden die Datenelemente der ICCR-Datensätze auf SNOMED CT abgebildet.

### Stand der Technik

**Abb. 1:**
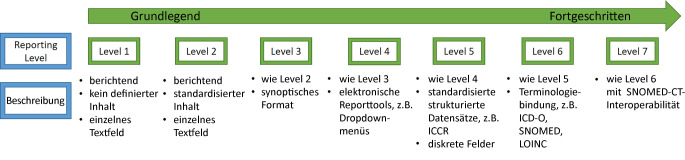
Die 6 Level des synoptischen Reportings (SR) und SNOMED CT als Level 7. (Mod. nach Ellis et al. [6])

Histopathologische Befunde haben unterschiedliche Level von Interoperabilität [6]. Das heißt, ihre maschinelle Verarbeitung führt zu unterschiedlichen Graden maschineller Interpretierbarkeit. Diese sind in Abb. 1 dargestellt:

1. Textbefunde ohne definierten Inhalt
2. Textbefunde mit (möglicherweise lokal) standardisiertem Inhalt
3. Synoptisches Format und Unterteilung in mehrere Textfelder – zum Beispiel Abschnitte Makroskopie und Mikroskopie und zusammenfassendes Gutachten
4. Nutzung von elektronischen Tools – zum Beispiel Dropdownmenüs und Checkboxen
5. Nutzung von standardisierten strukturierten Datensätzen – zum Beispiel ICCR-Datensätze
6. Nutzung von standardisierten Terminologien – zum Beispiel ICD‑O, TNM
7. Um die besondere Bedeutung von SNOMED CT hervorzuheben, wurde die Abbildung um ein Level 7 *Interoperabilität der Daten mittels SNOMED CT* ergänzt

Level 3 ist dabei das in Deutschland am weitesten verbreitete Level. Das höchste Qualitätsniveau bietet Level 6. Die für Level 4 erforderlichen technischen Vorarbeiten sind bereits breit verfügbar. So können Datensätze in verschiedenen Formaten, bspw. in HL7 FHIR als FHIR-Questionnaire, modelliert und mittels Softwarebibliotheken, sog. Renderer, wie beispielsweise LHC-Forms, in Laborinformationsmanagementsysteme (LIMS) integriert werden. Für Level 5 fehlen in Deutschland breit akzeptierte Datensatzdefinitionen. Die von der ICCR erarbeiteten Datensätze bieten hier zwar eine gute Grundlage, sind aber noch nicht auf Deutsch verfügbar. Für Level 6 existieren bereits eine Reihe von Terminologiesystemen, bspw. ICD-10, ICD‑O und TNM, die zu Krebsregistrierungszwecken ohnehin vorgeschrieben sind und daher bereits flächendeckend genutzt werden. Zur Steigerung der Interoperabilität der Daten ist hier eine Nutzung von SNOMED CT zu empfehlen, die in manchen Darstellungen bereits in Form von Level 7 explizit betont wird. Zur flächendeckenden Einführung dieses abschließenden Levels fehlen auch international noch weitere Vorarbeiten. So ist die Zuordnung der in den ICCR-Datensätzen vorgesehenen Datenelemente auf entsprechende SNOMED-CT-Codes nicht öffentlich verfügbar. In Deutschland ist eine Nutzung von SNOMED CT aufgrund der im Jahr 2021 erfolgten Lizensierung von SNOMED CT durch das BfArM jeder Pathologie in Deutschland ohne Mehrkosten möglich. Zugleich bemüht sich das BfArM in Zusammenarbeit mit diversen Medizinischen Fachgesellschaften und Aktivitäten um eine Usecase-basierte Übersetzung von englischsprachigen SNOMED-CT-Konzepten ins Deutsche. Eine vollständige Übersetzung von SNOMED CT ist aktuell allerdings nicht vorgesehen, sodass ergänzende Aktivitäten zur Übersetzung von für die Pathologie relevanten Konzepten notwendig sind.

## Methodik

**Abb. 2:**
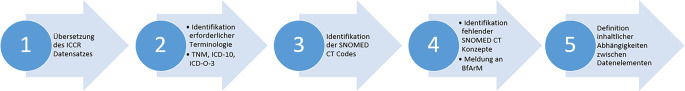
Prozessschritte zur Vorbereitung von ICCR-Datensätzen im Rahmen von synoptischem Reporting (SR) Level 6

Im Rahmen der vorliegenden Arbeit wurden die Limitationen des oben dargestellten Stands der Technik anhand des Usecase TUR‑B adressiert. Hieraus ergibt sich ein strukturierter Prozess, der für weitere ICCR-Datensätze wiederholt werden kann. Abb. 2 zeigt ein Flussdiagramm dieses Prozesses, der im Folgenden näher erläutert wird.

### Schritt 1.

Es erfolgt die Übersetzung des ICCR-Datensatzes. Der Datensatz enthält bereits die Definition von Pflichtfeldern, die für die Erstellung eines vollständigen Befundes erforderlich sind. *Appendix A* im Online-Supplement zeigt den übersetzten Datensatz zur TUR‑B in Form der von der ICCR erarbeiteten Eingabemaske.

### Schritt 2.

Dieser Schritt identifiziert die erforderlichen Terme aus den relevanten Terminologiesystemen. Das Mapping der Datenfelder des ICCR-Datensatzes auf die Codes von ICD-10, ICD‑O sowie TNM wird von der ICCR bereits vorgegeben. Im Rahmen nationaler Anpassungen, bspw. ICD-10-GM, sind hier jedoch gegebenenfalls Modifikationen vorzunehmen.

### Schritt 3.

Den dritten Schritt stellt die Identifikation von Codes für die Terminologiebindung dar. *Appendix B* im Online-Supplement listet als Beispielterminologie auf, welche SNOMED CT relevant sind und zu welchen Elementen des Datensatzes sie gehören. Die dortige Tabelle ergänzt dabei die Tabelle, die bereits Bestandteil der Definition des ICCR-Datensatzes ist und u. A. bereits die TNM-Klassifikation enthält. Neben der Abbildung auf bereits existierende SNOMED-CT-Konzepte wurden zusätzliche Konzepte identifiziert, die in SNOMED CT bisher nicht existieren oder nicht hinreichend präzise, für den Datensatz aber erforderlich sind. Diese betrifft im TUR‑B Datensatz die folgenden Konzepte:

- „Invasion superficial to muscularis mucosae“ fehlt.
- „Nested urothelial carcinoma (morphological abnormality)“ fehlt.
- „Plasmacytoid urothelial carcinoma (morphological abnormality)“ fehlt.

### Schritt 4.

Die Liste der im Rahmen des dritten Schrittes identifizierten Codes ist aufgrund der Existenz von Freitextfeldern im Datensatz unvollständig. Der potenzielle Inhalt dieser Felder könnte aber bspw. durch händische Strukturierung von Befundtexten aus dem Krebsregister identifiziert werden. Den Elementen dieser Liste können dann die entsprechenden SNOMED-CT-Codes zugeordnet werden. Die ICCR empfiehlt diesen Schritt explizit nicht, dennoch sollte er für künftige Anpassungen des Datensatzes berücksichtigt werden.

### Schritt 5.

Im Rahmen der Modellierung des Datensatzes ist es erforderlich, inhaltliche Abhängigkeiten zwischen den Datenelementen zu definieren. So sollte, bspw. im Rahmen des TUR‑B Datensatzes, die Eingabe eines pT1-Substaging nur dann möglich sein, wenn auch ein pT1 Tumor diagnostiziert wird. Die im Rahmen der Arbeit identifizierten wichtigen inhaltlichen Abhängigkeiten des TUR‑B Datensatzes listet Tab. 1 auf und erläutert diese kurz. Jede Zeile entspricht einer Abhängigkeit. Die Spalte Prämisse stellt dabei die inhaltliche Grundvoraussetzung dar, während die Spalte Konklusion die damit einhergehende Variablenbelegung bzw. Verpflichtung zur Angabe eines Datenelements zeigt. Im Sinne der Übersichtlichkeit werden rein technisch bedingte Abhängigkeiten, bspw. Spezifikation eines anderen histologischen Subtyps nur bei vorheriger Angabe „Andere“ beim histologischen Tumortyp möglich, ausgelassen. Zusätzlich zu den inhaltlichen Abhängigkeiten empfiehlt es sich, weitere Ausfüllhilfen, bspw. *Die Angabe eines eindeutigen Differenzierungsgrades ist gegenüber der Angabe ‚G3–G4‘ zu bevorzugen,* hinzuzufügen und diese aus der Definition des Datensatzes zu übernehmen.

**Tab. 1:** Auflistung der inhaltlichen Abhängigkeiten zwischen den Datenelementen des ICCR-Datensatzes transurethrale Blasenresektion (TUR-B)

Prämisse	Konklusion	Erläuterung
Histologischer Tumortyp = Urothelkarzinom	Histologische Subtypen/Subvarianten inkl. Anteile können angegeben werden	Angabe von Subtypen des Urothelkarzinoms nur bei Vorliegen eines Urothelkarzinoms sinnvoll
Ausmaß der Invasion = Tumorinvasion unter Einbeziehung des subepithelialen Bindegewebes (Lamina propria)	Substaging pT1 kann angegeben werden	Substaging pT1 kann nur bei pT1-Tumor bestimmt werden
(Proben‑)Entnahmelokalisation	Hinweis: Bei Proben von mehreren Lokalisationen bitte Formular je Lokalisation einmal ausfüllen	Die vorliegende Darstellung als Formular ist je Lokalisation einmal auszufüllen. Patienteninformationen müssen zwischen den Positionen gleich sein
Block-ID (Position)	Hinweis: Bei Blöcken aus mehreren Lokalisationen bitte Hinweis zur Lokalisation beachten	Dokumentation des Zusammenhangs zwischen klinisch angegebener Entnahmelokalisation und Proben-ID des Eingangsmaterials
Histologischer Tumorgrad	Hinweis: Bitte zuvor histologischen Tumortyp spezifizieren!	–
Invasionstiefe (T)	Hinweis: Die Angabe eines Substagings pT1a/pT1b ist gegenüber der reinen Angabe pT1 zu bevorzugen	Spezifischere Information ist allgemeinerer Information gegenüber grundsätzlich zu bevorzugen oder die Terminologie ist entsprechend zu ändern
Tumorgrad (G)	Die Angabe eines eindeutigen Grades ist gegenüber der Angabe „G3–4“ zu bevorzugen	Spezifischere Information ist allgemeinerer Information gegenüber grundsätzlich zu bevorzugen oder die Terminologie ist entsprechend zu ändern

Die Korrektheit der beschriebenen Prozessschritte wurde im Rahmen der Arbeit parallel durch das händische Strukturieren entsprechender Befunde unterstützt. Insbesondere für den ersten Schritt wurde der ICCR-Datensatz zwischen einer Pathologin und einem Informatiker diskutiert.

## Ergebnisse

Aus dem oben beschriebenen Prozess lässt sich eine Reihe von Empfehlungen ableiten, die im Folgenden diskutiert werden. Neben Empfehlungen für die Übersetzung weiterer Datensätze wird der nun verfügbare deutsche ICCR-Datensatz zur TUR‑B vorgestellt. Schließlich werden die zu empfehlenden Rahmenbedingungen zur Modellierung der Datensätze und der damit einhergehenden technischen Vorbereitung für die Nutzung der Datensätze in der Praxis aufgezeigt.

### Empfehlungen für den Datensatz

Basierend auf dem obigen Prozess sollte die deutsche Version eines ICCR-Datensatzes 2 Bestandteile haben:

1. ICCR-Datensatz ohne weitere Änderungen, lediglich Übersetzung,
2. Felder für die zu Krebsregistrierungszwecken erforderlichen Terminologien: ICD-10, ICD‑O und TNM.

Weitere inhaltliche Anpassungen sollten im Sinne der Standardisierung des Datensatzes nicht vorgenommen werden. Im Rahmen dieser Arbeit wurde ein einzelner Datensatz übersetzt und für SR vorbereitet. Bei der Übersetzung weiterer Datensätze sollte auf eine zwischen den Datensätzen konsistente Übersetzung geachtet werden, um später eine intuitive Dateneingabe zu erleichtern. Eine Abbildung der Datenelemente des Datensatzes auf SNOMED CT sollte parallel zur Übersetzung des Datensatzes erfolgen, um eine möglichst frühzeitige flächendeckende Nutzung von SNOMED CT in Deutschland zu fördern. Somit besteht die Möglichkeit, die erforderlichen SNOMED-CT-Konzepte ebenso zu übersetzen, dem BfArM zu melden und somit der Allgemeinheit zur Verfügung zu stellen.

### Empfehlungen für die Modellierung des Datensatzes

Der hiermit nun vorliegende deutsche ICCR-Datensatz zur TUR‑B steht für die Nutzung grundsätzlich zur Verfügung. Zur vereinheitlichten Nutzung des Datensatzes ist es zu empfehlen, dass die Modellierung in einem Standardformat, bspw. als FHIR-Questionnaire, zentral vorgenommen wird. Die dabei entstehende Modellierung kann dann öffentlich verfügbar gemacht und durch die LIMS-Entwickler leicht in die bestehenden Systeme integriert werden. Da der ICCR-Datensatz bereits über definierte Pflichtfelder verfügt, können diese in die Modellierung übernommen werden, um ein unvollständiges Ausfüllen der Maske zur Datenerfassung zu verhindern. Im Sinne der Datenqualität sollten die bereits identifizierten inhaltlichen Abhängigkeiten in der Modellierung berücksichtigt werden. Dies sollte durch die direkte Einbettung der identifizierten SNOMED-CT-Codes ergänzt werden. Somit entsteht bei der Dateneingabe kein Mehraufwand für die Darstellung der Daten in SNOMED CT. Sprachlich bietet sich für Eingabeaufforderungen im Rahmen der Modellierung die Formulierung „Bitte plus Infinitiv“ – bspw. *Bitte spezifizieren* – an.

## Diskussion

Der nun vorliegende deutsche ICCR-Datensatz zur TUR‑B steht nun öffentlich für die Nutzung zur Verfügung. Die zugehörigen SNOMED-CT-Konzepte sind ebenfalls öffentlich verfügbar. Somit sind die Vorbereitungen geschaffen, um Level 6 des SR zu erreichen:

1. Nutzung von elektronischen Tools kann durch Modellierung, zum Beispiel als FHIR-Questionnaire, erreicht werden.
2. Mit dem ICCR-Datensatz wird ein standardisierter Datensatz genutzt, der nun auch Belange der Abrechnung berücksichtigt.
3. Mit ICD‑O, ICD-10 und TNM werden standardisierte Terminologien genutzt. Die Interoperabilität der Daten wird mittels SNOMED CT unterstützt.

Der Methodenteil des vorliegenden Artikels zeigte einen strukturierten Prozess, der für die Etablierung weiterer deutscher ICCR-Datensätze wiederholt werden kann. Eine Nutzung von über die reine Codierung von Befundinhalten hinausgehenden Features von SNOMED CT bietet weiteres Potenzial zur Qualitätsverbesserung der Diagnostik. So könnten automatisiert Querverbindungen zu anderen potenziellen Diagnosen gezogen werden und die dahinterstehenden Feststellungen können in die Erstellung des Pathologiebefundberichts einfließen.

## Ausblick

Die Übersetzung der ICCR-Datensätze einschließlich deren Aktualisierungen sollten in einem strukturierten Prozess erfolgen, der im vorliegenden Artikel beschrieben wurde. Zur Vermeidung von Parallelentwicklungen sollte die Übersetzung und Anreicherung der Datensätze mit SNOMED-CT-Codes zentral koordiniert werden – bspw. durch die Aktivitäten der AG Semantik des Berufsverbands Deutscher Pathologen koordiniert durch die Qualitätsinitiative Pathologie. In diesen Prozess sollten Pathologen, Kliniker und Informatiker einbezogen werden. Am Ende des Prozesses sollte jeder Datensatz, zum Beispiel als FHIR-Questionnaire, modelliert sein und den LIMS-Herstellern verfügbar gemacht werden.

Die im Zuge des Prozesses identifizierten SNOMED-CT-Codes können im Rahmen der Übersetzung des Datensatzes ebenfalls übersetzt und anschließend dem National Release Center for SNOMED beim BfArM gemeldet werden. Gemeldet werden sollte dort ebenfalls, wenn Konzepte in SNOMED CT fehlen. So leistet die deutsche Pathologie gleichzeitig einen Beitrag zur internationalen Weiterentwicklung.

In Ergänzung zur abstrakten Abhandlung der Datensätze sollte die konkrete Nutzung der modellierten Datensätze einem Praxistest unterzogen werden. Zu betrachten ist hier insbesondere, inwiefern sich das Arbeiten der Ärzte hier verändert. Zu Beginn ist ein zeitlicher Overhead zu erwarten, da die Dateneingabe statt als Diktat nun über Maus und Tastatur erfolgt. Auch könnte es einen Unterschied machen, wenn im Rahmen der Mikroskopie noch ein klassisches Mikroskop zum Einsatz kommt. Im Falle des Diktats musste der Arzt bzw. die Ärztin bisher nicht vom Mikroskop aufblicken, um Daten einzugeben. Bei der Nutzung von SR über eine klassische graphische Benutzeroberfläche ist dies jedoch erforderlich. Eine mögliche Lösung hierfür kann die Nutzung von AI-basierten Systemen zur Übertragung des gesprochenen Textes in die Eingabemaske mit anschließender Prüfung durch den Arzt bzw. die Ärztin sein. Zumindest theoretisch sind solche Systeme bereits verfügbar: OpenAI bietet im Rahmen von ChatGPT (OpenAI OpCo LLC, San Francisco, CA, U.S.) sowohl die Möglichkeit einer Spracheingabe als auch die Möglichkeit, Daten aus Text strukturiert auszugeben. Der produktive Einsatz eines solchen Ansatzes dürfte derzeit im Falle von (Chat‑)GPT an den regulatorischen Rahmenbedingungen und im Falle von Open-Source-Modellen an deren Performance scheitern. Betrachtet man die derzeitige Entwicklung von vergleichbaren Large-Language-Modellen, so sind proprietäre Modelle wie GPT Open-Source-Modellen um einige Zeit voraus. Wir dürfen daher – zumindest im Hinblick auf die reine Performance der Modelle – in den kommenden Jahren mit der öffentlichen Verfügbarkeit von hinreichend performanten Modellen rechnen.

Als positive Folge der Einführung von SR ist im Zusammenhang mit der weiteren Verbreitung der digitalen Pathologie von einem zusätzlichen Synergieeffekt auszugehen. Denkbar wäre hier unter anderem eine Integration der Eingabemaske in das digitale Mikroskop. So könnte im Rahmen der Annotation des Schnittes. zum Beispiel hinsichtlich des tiefsten Invasionspunktes des Karzinoms, direkt das pT-Staging ausgewählt werden.

In weiteren Schritten sollten nun die Übersetzung der ICC- Datensätze ins Deutsche sowie die Modellierung von Erkrankungen mittels SNOMED CT vorangetrieben werden. Hierzu sind zentral koordinierte Aktivitäten erforderlich, um einer weiteren Ausweitung der Heterogenität der Befunddokumentation der deutschen Pathologien vorzubeugen.

## Fazit für die Praxis

- Die Schritte der vorliegenden Arbeit sollten für die weiteren ICCR-Datensätze wiederholt werden
- Pathologien sollten keine eigenen Anpassungen an den Datensatz vornehmen, sondern bereits modellierte Datensätze nutzen. Diese sollten versioniert werden, sodass Sie bei Änderungen des ICCR-Datensatzes oder der verwendeten Terminologie angepasst werden können. Relevant wird dies zum Beispiel im Rahmen der flächendeckenden Einführung von ICD-11.
- Bei der Anschaffung von Laborinformationsmanagementsystemen (LIMS) sollte darauf geachtet werden, dass das LIMS die Eingabe und Verwaltung strukturierter Befunde unterstützt

## Supplementary Information

Appendix 1 enthält das ins Deutsche übersetze Formular der ICCR für den Datensatz zur Transurethralen Resektion der Harnblase (TUR-B; vgl. Abschnitt Methodik Schritt 1). Appendix B enthält das Mapping der im ICCR Datensatz TUR-B vorgesehenen Elemente auf SNOMED CT (vgl. Abschnitt Methodik Schritt 3).

## Data Availability

Im Rahmen der Arbeit wurden keine neuen Daten erhoben.
