# Supplementary material for: Establishment of a German ICCR dataset: Translation and integration of SNOMED CT using the example of TUR-B
Source: Pathologie (Heidelb). 2024 Dec 5;46(2):108–14. [Article in German] doi: 10.1007/s00292-024-01398-3 (PMC11861236; doi:10.1007/s00292-024-01398-3)
Supplement: Supplementary file 1 — Appendix 1 enthält das ins Deutsche übersetze Formular der ICCR für den Datensatz zur Transurethralen Resektion der Harnblase (TUR-B; vgl. Abschnitt Methodik Schritt 1). Appendix B enthält das Mapping der im ICCR Datensatz TUR-B vorgesehenen Elemente auf SNOMED CT (vgl. Abschnitt Methodik Schritt 3). [file 292_2024_1398_MOESM1_ESM.docx]

# APPENDIX A

**Befundleitaden für Karzinome des Urinaltraktes im Rahmen von Biopsien und transurethralen Resektionen**

Nachname Geburtsdatum

Vorname

Fallnummer Eingangsdatum Eingangsnummer

| **Fett** gedruckte Elemente sind VERPFLICHTEND. Elemente in **grau** werden EMPFOHLEN. **KLINISCHE ANGABEN**  **Vorerkrankungen des Urogenitaltraktes oder Fernmetastasen**  Keine Angaben Keine Vorerkrankung  Details einschließlich Lokalisation    **Vorangegangene Therapien**  Keine Angaben Keine vorangegangene Therapie  Wenn ja, dann bitte Art der Therapie angeben  **Cystoskopisches Erscheinungsbild** (wähle alle zutreffenden)  Keine Angaben  Papillär  Polipös  Roter (erythomatöser) Bereich  Anderes, bitte spezifizieren  Normal   \|  \| \| --- \|   Weitere klinische Angaben, bitte spezifizieren  **(PROBEN-)ENTNAHMELOKALISATION***  Renal pelvis              Ureter  Blase, bitte genaue Lokalisation angeben  Prostate/prostatic urethra  Urethra*, specify site(s)*  Andere, bitte spezifizieren    * *If biopsies are from different locations then a separate dataset should be completed for each specimen site.*  **CHIRURGISCHES VORGEHEN**   \|  \| \| --- \|   Keine Angabe          Transurethrale Resektion (TUR)  Biopsie  Andere, bitte spezifizieren  **ASSOZIIERTE EPITHELIALE LÄSIONEN**  *Vorhanden, bitte spezifizieren*  *Nicht identifiziert*  **HISTOLOGISCHER TUMORGRAD**  Low-Grade  High-Grade  Andere, bitte spezifizieren   \|  \| \| --- \|   **Plattenepithelkarzinom oder Adenokarzinom**  GX: Nicht beurteilbar  G1: Hochgradig differenziert  G2: Mäßiggradig differenziert  G3: Geringgradig differenziert  Andere, bitte spezifizieren   \|  \| \| --- \| | **BLOCK-ID (POSITION)**  **HISTOLOGISCHER TUMORTYP**  *(Value list from the WHO Classification of Tumours of the*  *Urinary System and Male Genital Organs (2016))*    Urothelkarzinom    Plattenepithelkarzinom    Adenokarzinom    Tumore vom Müllerschen Typ    Klarzelliges Karzinom    Endometroides Karzinom    Neuroendokrine Tumore    Kleinzelliges neuroendokrines Karzinom    Großzelliges neuroendokrines Karzinom    Andere, bitte spezifizieren    **Histologischer Subtyp / Variante (Urothelkarzinom)**  Nicht identifiziert  Vorhanden, bitte Subtyp und prozentualen Anteil spezifizieren  (Bitte alle Zutreffenden wählen)  Plattenepithel 🡪 %  Drüsig 🡪 %  Nested 🡪 %  Mikropapilär 🡪 %  Plasmazytoid / Diffus 🡪 %  Sarkomatoid 🡪 %  Andere, bitte spezifizieren  **🡪** %  **NICHT-INVASIVE KARZINOME** (wähle alle zutreffenden)  Nicht identifiziert  Nicht bestimmbar  Carcinoma in-situ, flach  Unifokal Multifokal  Papilläres Karzinom, nicht invasiv  Andere, bitte spezifizieren   \|  \| \| --- \| |
| --- | --- | --- | --- | --- | --- | --- |
| **NACHWEIS VON MUSCULARIS PROPRIA**  Vorhanden Nicht vorhanden/übermittelt    **AUSMAß DER INVASION** (wähle alle zutreffenden)  Nicht beurteilbar  Papilläres Karzinom, nicht-invasiv  Carcinoma in-situ, flach  Tumorinvasion unter Einbeziehung des subepithelialen Bindegewebes (Lamina Propria)  Tumorinvasion unter Einbeziehung der Muscularis Propria (Detrusormuskel)  Tumorinvasion unter Einbeziehung des prostatischen Stromas  **SUBSTADIUM ZU pT1**   \| mm \| \| --- \|   Tiefe der Invasion  UND/ODER   \| mm \| \| --- \|   Absolute maximale Dimension des invasiven Tumors  UND/ODER  Invasion oberhalb der Muscularis Mucosae  Invasion unter Inanspruchnahme und/oder tief in die Muscularis Mucosae | **LYMPHOVASKULÄRE INVASION**  Nicht identifiziert Vorhanden  Nicht bestimmbar  **NEBENBEFUND**  Keine identifiziert  Identifiziert, bitte spezifizieren  **ZUSATZUNTERSUCHUNGEN**  Nicht durchgeführt  Durchgeführt, bitte spezifizieren |

# APPENDIX B

| Element Name | Value -> SNOMED CT code |
| --- | --- |
| SPECIMEN SITE | • Bladder, specify site(s) -> In hierarchy below 89837001  • Other, specify -> In hierarchy below 122489005 |
| OPERATIVE PROCEDURE | • Transurethral resection (TUR) ->287708003  • Biopsy -> 36503003 |
| HISTOLOGICAL TUMOUR TYPE | • Urothelial carcinoma -> 27090000  • Squamous cell carcinoma -> 1162767002  • Adenocarcinoma -> 1187332001  • Tumours of Müllerian type  o Clear cell carcinoma ->30546008  o Endometrioid carcinoma -> 30289006  • Neuroendocrine tumour  o Small cell neuroendocrine carcinoma -> 719105002  o Large cell neuroendocrine carcinoma -> 128628002  • Other, specify -> In hierarchy below 1240414004  Histological sub-type/variant (urothelial carcinoma):  • Not identified -> 47492008  • Present: -> 52101004  o Squamous -> 399464005  o Glandular -> 399564007  o Micropapillary -> 128639004  o Plasmacytoid -> 1208461001  o Sarcomatoid -> 1208484008 |
| NON-INVASIVE CARCINOMA | Single selection value list:  • Not identified -> 47492008  • Indeterminate -> 404684003  • Carcinoma in situ, flat -> 396199003  • Papillary carcinoma, non-invasive -> 128877008 |
| ASSOCIATED EPITHELIAL LESIONS | Single selection value list:  • Present, specify -> In hierarchy below 396663007  • Not identified -> 396664001 |
| HISTOLOGICAL TUMOUR GRADE | Single selection value list:  • Not applicable -> 385432009  • Cannot be determined -> 384741006  Urothlial carcinoma:  • Low-grade -> 395529007  • High-grade -> 395530002  Squamous cell carcinoma or adenocarcinoma  • GX: Cannot be assessed -> 1155705000  • G1: Well differentiated -> 1155701009 • G2: Moderately differentiated -> 1155703007 • G3: Poorly differentiated -> 1155704001  • Other, specify -> 1155702002 (G4) |
| STATUS OF MUSCULARIS PROPRIA | Single selection value list:  • Present -> 396662002  • Not present/submitted -> 396661009 |
| EXTENT OF INVASION | • Cannot be assessed -> 370048007  • Papillary carcinoma, non-invasive -> 128877008  • Carcinoma in situ, flat -> 1187138006  • Tumour invades subepithelial connective tissue (lamina propria) -> 395533000  • Tumour invades muscularis propria (detrusor muscle) -> 370060008  • Tumour invasive into prostatic stroma -> 399433008 |
| SUBSTAGING T1 DISEASE | • Invasion involving and/or deep to muscularis mucosae -> 395534006 |
| LYMPHOVASCULAR INVASION | • Not identified -> 47492008  • Present -> 1155929004  • Indeterminate -> 395720009 |
| COEXISTENT PATHOLOGY | • None identified -> 47492008  • Present, specify -> In hierarchy below 300457003, except for concepts below 126885006 |
